# Multi-scale patterns of tick occupancy and abundance across an agricultural landscape in southern Africa

**DOI:** 10.1371/journal.pone.0222879

**Published:** 2019-09-20

**Authors:** Kimberly J. Ledger, Ryan M. Keenan, Katherine A. Sayler, Samantha M. Wisely

**Affiliations:** 1 Department of Wildlife Ecology and Conservation, University of Florida, Gainesville, Florida, United States of America; 2 Department of Fisheries, Wildlife, and Conservation Biology, University of Minnesota, St. Paul, Minnesota, United States of America; Onderstepoort Veterinary Institute, SOUTH AFRICA

## Abstract

Land use influences the prevalence and distribution of ticks due to the intimate relationship of ticks with their environment. This relationship occurs because land use alters two essential tick requirements: vertebrate hosts for blood meals and a suitable microclimate when off-host. Given the risks to human and animal health associated with pathogens transmitted by ticks, there is an ongoing need to understand the impact of environmental drivers on tick distributions. Here, we assessed how landscape features, neighborhood effects, and edges influenced tick occupancy and abundance across an agricultural landscape in southern Africa. We found that *Rhipicephalus appendiculatus* and *Rhipicephalus simus* increased in abundance closer to protected savanna, while *Haemaphysalis elliptica* increased in abundance closer to human habitation. The composition of the landscape surrounding savanna patches also differentially influenced the occupancy of each tick species; *H*. *elliptica* was more likely to be found in savanna patches surrounded by subsistence agriculture while *R*. *appendiculatus* and *R*. *simus* were more likely to be found in savanna surrounded by sugarcane monocultures. At the local scale we found that *R*. *appendiculatus* and *R*. *simus* avoided savanna edges. The availability of hosts and variation in vegetation structure between commercial agriculture, subsistence agriculture, and savanna likely drove the distribution of ticks at the landscape scale. Understanding how anthropogenic land use influences where ticks occur is useful for land use planning and for assessing public and animal health risks associated with ticks and tick-borne diseases.

## Introduction

Indirectly transmitted pathogens, those that require an intermediate host or vector for transmission, are among the most sensitive pathogens to global change [1]. Anthropogenic forces, such as agricultural intensification, deforestation, and urbanization affect the occurrence of vector-borne diseases in humans and animals [1] and rank among the most cited drivers of zoonotic disease emergence over the past half century [2, 3]. Ticks are second worldwide to mosquitoes as the most important vectors of human pathogens, and are the most important vectors of disease-causing pathogens in domestic and wild animals [4]. With portions of their life cycle occurring both on-host and in the environment, ticks are impacted by changes in the landscape that alter host community composition as well as changes that modify the physical environment.

One such landscape change that alters the composition of the physical and biotic environment is conversion to intensive agriculture. The expansion of agricultural land is one of the most significant human modifications to the global environment [5] and one of the main drivers of biodiversity declines worldwide [6]. In rural regions of southern Africa, as in other developing regions of the world, large-scale, intensive, monoculture agriculture is replacing small-scale, heterogeneous, rain-fed agriculture [7] which leads to an overall simplification of the environment and reduction in total biodiversity. Specifically, crop homogenization, as part of the intensification process, alters the vegetation and microclimate that is crucial for tick survival when off-host, and causes declines in wildlife species that may serve as tick hosts through the direct loss of habitat [8].

The occurrence of ticks depends in part on the availability of vertebrate hosts. Ticks do not feed equally on all vertebrate animals and typically display some degree of host specificity [9]. Tick species can range from highly host-specific, including ticks with documented records from a limited number of hosts, to host generalists, including ticks with documented records from 20 or more different host species [10]. The Lowveld region of southern Africa supports many tick species that represent the gamut of host specialization (Table 1). For example, all life stages of *Haemaphysalis elliptica* (yellow dog tick) primarily fed on domestic dogs and wild carnivores [10, 11] but do not feed on other vertebrate animals. However, many ticks feed on only specific host species during one or two stages of their life cycle, but are less host specific in another stage of the life cycle, such as *Rhipicephalus simus*. Finally, generalist ticks, such as *Rhipicephalus appendiculatus* fall at the opposite end of the host specificity spectrum and feed on virtually any available host at each life stage. Therefore, the degree of host specificity of ticks and the distribution of hosts interact to influence the occurrence and abundance of ticks on the landscape.

**Table 1:** Selected list of known host species and tick-borne pathogens for ticks encountered at the study site in the lowveld of Eswatini.

Life Stage	Host Preferences	Pathogens (Disease)
***Rhipicephalus appendiculatus***		
larvae	*Domestic*: cattle, sheep, goats	**Livestock:**
	*Wild*: African buffalo, kudu, antelope species, waterbuck, Vervet monky,	*Theileria parva parva* (East Coast fever)^[12]^
	plains zebras, warthog, scrub hare	*Theileria parva lawrencei* (Corridor disease)^[13]^
		*Theileria parva bovis* (January disease)^[14]^
nymph	*Domestic*: cattle, dogs, sheep, goats	*Theileria taurotragi* (benign theileriosis)^[15]^
	*Wild*: African buffalo, kudu, antelope species, waterbuck, smaller	*Anaplasma bovis* (bovine anaplasmosis)^[16]^
	antelope species, plains zebras, warthog, scrub hare	*Nairovirus* (Nairobi sheep disease)^[17]^
		*Flavivirus* (Louping ill)^[18]^
adult	*Domestic*: cattle, dogs, sheep, goats	**Human:**
	*Wild*: African buffalo, kudu, antelope species, waterbuck, lions	*Rickettsia aeschlimanni* (Spotted fever)^[19, 20]^
***Rhipicephalus simus***		
larvae	*Domestic*: none	**Livestock:**
	*Wild*: Red veld rats, scrub hares, lion	*Theileria parva parva* (East Coast Fever)^[21]^
nymph	*Domestic*: none	*Anaplasma marginale* (bovine anaplasmosis)^[22]^
	*Wild*: Red veld rats, scrub hares	*Anaplasma centrale* (bovine anaplasmosis)^[23]^
adult	*Domestic*: Dogs, cattle, sheep, goats	**Human:**
	*Wild*: lions, common warthogs, cheetahs, leopards, African buffalo,	*Rickettsia conorii* (Mediterranean spotted fever)^[24]^
	kudu, impala, plains zebra, rhinoceros, humans	
***Haemaphysalis elliptica***		
larvae	*Domestic*: Dogs, cats, goats	**Dog:**
	*Wild*: rodents, yellow mongoose	*Babesia canis* (canine babesiosis)^[25]^
nymph	*Domestic*: Dogs, cats	**Human:**
	*Wild*: rodents, yellow mongoose	*Rickettsia conorii conorii* (Mediterranean spotted
adult	*Domestic*: Dogs	fever)^[26, 27]^
	*Wild*: lion, leopard, African civet, African wild dog, jackals	
***Amblyomma hebraeum***		
larvae	*Domestic*: cattle, goats	**Livestock:**
	*Wild*: similar to adults, as well as carnivores, small antelopes, scrub	*Ehrlichia ruminantium* (heartwater)^[28]^
	hares, and game birds, lions, cheetahs	large wounds from long mouthparts^[11]^
	*not rodents	**Human:**
nymph	*Domestic*: cattle, goats	*Rickettsia africae* (African tick-bite fever)^[29]^
	*Wild*: similar to adults, as well as carnivores, small antelopes, scrub	
	hares, and game birds, lions, cheetahs	
	*not rodents	
adult	*Domestic*: cattle, goats	
	*Wild*: large ruminants (such as giraffes, buffalo, elands, kudu), warthogs	

Selected list of common domestic and wildlife host preferences and known pathogens vectored by ticks detected in northeastern Kingdom of Eswatini in June and July 2017. *Rhipicephalus appendiculatus*, adult *R*. *simus*, and *Amblyomma hebreaum* are generalist ticks, while *Haemaphysalis elliptica* is a carnivore specialist. Known pathogens are grouped by the taxa in which they cause disease. Host preference citations are reviewed in [10, 11].

Characteristics of the habitat, such as vegetation composition, in which ticks and their hosts live, directly affect the microclimate conditions available to ticks. An appropriate microclimate, which encompasses various small-scale climatic conditions near the ground surface such as temperature, wind speed, exposure, saturation deficit, and soil moisture [30], are necessary for the survival for ticks when off host. Experimental laboratory studies have shown that air temperature and relative humidity affect the survival of many tick species [31–33]. In general, survival of all life stages generally declines with increasing temperature and decreasing relative humidity. Ground vegetation may buffer ticks from the extreme fluctuations in their microclimatic conditions. For example habitats with greater shrub cover, denser understories, and greater canopy cover have shown higher tick abundances [34–36]. Studies in Africa found that ticks use habitats that provide cover from high temperatures and provide cooler, humid environments [30, 37, 38], however some tick species may be adapted to hot and dry conditions, as is the case for *Rhipicephalus zambesiensis* [39]. In general, land use change can alter both the potential host species for ticks in an area and the microclimate.

In addition to directly influencing the vertebrate host and vegetative composition, agricultural intensification can change the configuration of the landscape in ways that that may further impact tick distribution. At the landscape level, land use patterns and features, such as the proximity and connectivity of land covers that contain appropriate host species or influence host movements, can affect the occurrence of ticks across a landscape [40]. For example, the creation of ecotones and woodland cover due to agricultural expansion in rural France promoted increased *Ixodes ricinus* abundance by increasing host populations that were suited to these land use changes [41]. Yet in the United States, studies have found conflicting effects of fragmentation on the abundance of *I*. *scapularis* (e.g. [42, 43]) suggesting that local context of host habitat use and scale of the study play a large role on the cascade of effects to parasites and their pathogens.

Drivers of vector abundance and disease risk appear to be scale dependent. In addition to landscape scale drivers such as edge patch ratios, landscape heterogeneity, and landscape composition, neighborhood effects such as the composition of adjacent habitat patches can influence species in a focal patch [44]. Within a patch, tree species composition [45], shrub density [46] and proximity to the patch edge [47] can influence tick abundance. These drivers can proximally affect the diversity [48] and density [36] of suitable hosts, or the suitability of microhabitat conditions necessary for off-host tick survival [49]. In the Lowveld region of southern Africa, monoculture agriculture in the form of sugarcane is rapidly replacing rain-fed agriculture and savanna ecosystems [7] which alters host dynamics [50] and microclimate features, yet has unknown consequences to tick ecology.

In this investigation, we examined the influence of the environment on tick occupancy and abundance at three spatial scales: landscape (defined by a buffer zone surrounding sampling site), neighborhood (defined as the local region which influences the organism and parameterized as the land cover class of the sampled patch and adjacent patch), and patch scale (defined as the sampling location, the region of a continuous single landcover class in which the sampling transects occurred). We used the presence or number of questing ticks to estimate occupancy or relative abundance. By surveying for questing ticks we define a measure that directly influences the risk of humans or animals being parasitized by ticks. At the landscape scale, we predicted lower risk of parasitism from ticks which utilize large wildlife hosts (such as adult *R*. *simus* and all stages of *R*. *appendiculatus*) as distance from protected savanna increases and the proportion of native savanna landcover decreases. Alternatively, we predicted higher risk of parasitism from ticks which utilize domestic hosts (such as the canid specialist tick *H*. *elliptica)* closer to human habitation and subsistence agriculture. These predictions were based on our assumption that protected areas have higher densities of large and medium wildlife species compared to the surrounding areas due to improved habitat quality and reduced poaching intensity [51]. By contrast, we assumed the proximity to community settlements was positively associated with domestic host species such as cattle, goats, chickens and dogs [52].

At the neighborhood scale, we predicted that since monoculture crop simplifies the environment, reduces ground cover and is inhospitable to large ungulates, there would be lower risk of tick parasitism in sugarcane agriculture than in subsistence agriculture or in savanna. Additionally, we predicted the composition of neighboring habitat would influence the tick species in the focal habitat by providing habitat for hosts that occasionally utilize the focal habitats, such as the domestic animals associated with subsistence agriculture that occasionally enter the surrounding savanna. At the patch scale, we hypothesized a higher risk of tick parasitism would be associated with savanna edge habitat due to an increase in number of potential hosts or a higher density of suitable hosts near edges.

## Materials and methods

### Study area

**Fig 1:**
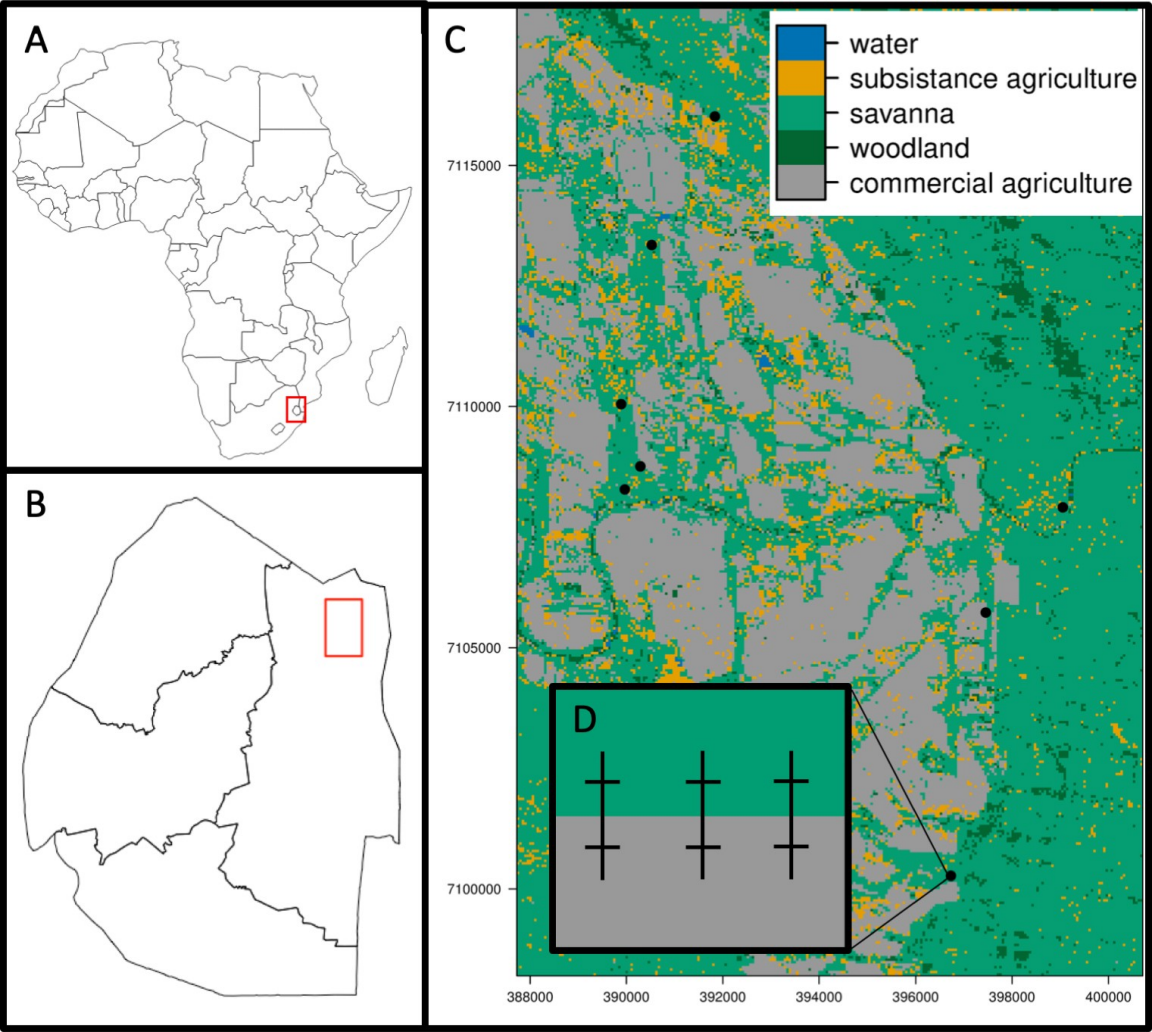
Map of study area and sampling design. A) Map of Africa showing the location of the Kingdom of Eswatini inside the red box. Image from Natural Earth (http://www.naturalearthdata.com/) B) Map of the Kingdom of Eswatini showing the extent of the study area inside the red box. Image from Natural Earth (http://www.naturalearthdata.com/) C) Land-cover classifications [50] and the sampling locations (black points) within the study area. Land-cover classes included commercial agriculture in red, subsistence agriculture in gray, savanna in light green, woodland in dark green and water in blue. D) A typical site containing three 200-m transects (dashed lines) that extend 100-m into each land-cover (here: savanna and sugarcane). One of the ten 20-m sub-transects for tick collection is shown in each land-cover perpendicular to the transect.

Our study occurred within the Lowveld savanna region in the northeastern portion of the Kingdom of Eswatini in southern Africa (Fig 1). This region experiences mild, dry winters (8-26°C; 0-50mm rainfall) and hot, wet summers (15-33°C; 200-500mm rainfall) [53]. The northeastern portion of the Kingdom of Eswatini mainly consists of government- and privately-owned protected areas [54], commercial agriculture in the form of intensive, irrigated sugarcane monocultures [55], and rural settlements surrounded by rain-fed subsistence agriculture [56]. Between 1990 and 2015, 48% of the Kingdom of Eswatini landcover changed due to the expansion of irrigated and fertilized sugarcane cropland and conversion of savanna to cropland [57]. This landcover change resulted in fragmentation of savanna outside of conservation areas.

Our approximately 10 km by 17 km study area (extent: S 26.2140 to S 26.0677; E31.8971 to E31.9906) included a patchwork of fragmented savanna, commercial agriculture (hereafter: sugarcane), and homesteads with subsistence agriculture. We obtained permission to sample inside multiple sugarcane estates owned by the Royal Swaziland Sugarcane Cooperation. Each sugarcane estate included large plantations of irrigated sugarcane, roads, water storage, canal systems, offices and maintenance workshops. Only mature (greater than three meters tall) sugarcane monoculture patches were included in this study. We obtained permission to sample in and around homesteads from area chiefs and homeowners. Homesteads with subsistence agriculture consisted of families living in several homes, and often contained dogs, poultry, and ruminant livestock. The vegetation surrounding homesteads was subsistence agriculture patches and included rain-fed cultivated plants such as corn, sweet potato, cassava, and beans. The vegetation in the fragmented savanna patches was characterized as *Acacia* savanna [58], and consisted of native trees and grasses. Species diversity of vegetation was highest in savanna, followed by communal land, and then the predominately sugarcane monoculture. Bare ground was lower in savanna regions compared to sugarcane [59], with the least amount of vegetation occurring in communal lands. The conservation areas adjoining our study area, Hlane Royal National Park and Mbuluzi Game Reserve, were characterized as *Acacia* savanna, broadleaved woodland, and riverine forest [58]. The fauna of protected areas were composed of wild ungulates such as impala (*Aepyceros melampus*), blue wildebeest (*Connochaetes taurinus*), plains zebra (*Equus quagga burchellii*), bushpig (*Potamochoerus larvatus*), and giraffe (*Giraffa giraffa*) [60]. These and other wildlife species were largely restricted to protected areas due to hunting and poaching outside of these areas [51]. Thus the landscape of our study area was composed of three distinct habitat types (savannas, subsistence agriculture, and sugarcane monocultures) that allowed us to investigate the impact of landscape level processes such as how proximity to habitat types, including protected areas, and landscape composition affected tick communities.

### Tick flagging surveys

Certain species and life stages of free-living ticks can be sampled while exhibiting questing behavior. Questing ticks are collected by dragging, flagging, or walking through vegetation, or by using CO_2_ baited traps [9]. Flag sampling only surveys a portion of the tick community, because only a fraction of species and life stages quest in the vegetation. Nonetheless, many studies have compared occurrence or relative densities of free-living, questing ticks between sites by comparing the quantity of ticks collected per unit time or per unit distance (e.g. [61, 62]), and estimates of relative tick densities from flagging samples are commonly used as a measure of entomological risk (e.g. [63, 64]). In this study, we used flagging to compare occupancy and risk of encountering questing ticks between land cover classes. We assumed that any collection biases were consistent across sites.

Sampling occurred during the austral winter season from 14 June 2017 until 25 July 2017. Sites ranged from 165 meters to 312 meters in elevation. Tick collections were performed between 0700 and 1100, when the vegetation was typically dry and optimal for tick questing behavior. Studies on seasonal occurrence of ticks in southern Africa have recorded all life stages of ticks during this sampling period [10]. To assess tick communities at three spatial scales, we surveyed eight sites within the agricultural matrix of our study system in the Kingdom of Eswatini. Each site was centered on the border of two patches differing in land cover classification. Sites either consisted of a savanna patch next to a sugarcane patch (n = 4), or a savanna patch next to a subsistence agriculture patch (n = 4). Ticks were collected at three paired replicate subplots per site. Each pair of subplots consisted of ten 20 m transects each in savanna and in the alternate habitat (sugarcane or subsistence agriculture).

For each transect, a one-meter square white flannel cloth was flagged through the vegetation parallel to the interface of the two habitat types, with the first transect at the habitat edge (defined by a dirt or grass road), and each subsequent transect further extending into the habitat and spaced 10m apart (Fig 1). Flannel cloths were inspected every two meters and ticks found on the cloth were immediately placed in 90% molecular grade ethanol. In total, 240 20-m drag intervals occurred in fragmented savanna, 120 20-m drag surveys occurred in sugarcane, 120 20-m drag surveys occurred in subsistence agriculture. Sampling at each site was conducted for three consecutive days to account for imperfect detection. Additionally, time, temperature, and relative humidity were recorded at the start of 116 out of 144 transect surveys. For each transect missing survey-specific covariate data, the site average for each covariate was used. Dew point was calculated using temperature and relative humidity. Adult ticks were subsequently identified to species, while nymphs were identified to genus using microscopy and a standard key [11, 65]. Larval ticks were not identified.

We used spline correlograms and the Moran’s I statistic to estimate the autocorrelation of tick abundance among sites as a function of distance between sites. We tested for independence of sites (n = 8) by computing Moran’s I global statistic with 999 random Monte Carlo permutations [66] at sequential distance lags and visualized potential spatial autocorrelation using a spline correlogram with 100 bootstrapped samples for the 95% pointwise confidence interval [67].

We determined the scale at which landcover influenced tick occurrence using a multi-scale modeling approach. A common procedure to identify the scale of effect is by establishing multilevel buffers around sampling locations to detect species’ responses at several scales [68]. We used a ~50m by ~50m resolution landcover map derived from Google Earth Engine [50] to characterize the landscape. This map included five land-cover classes (sugarcane, subsistence agriculture, savanna, woodland, and open water) that spanned the Lowveld savanna region of the Kingdom of Eswatini. The landcover map was aggregated to ~100m by ~100m resolution to match the resolution of landcover map to the size of the sampled transects. We calculated the proportion of three focal landcovers (savanna, sugarcane and subsistence agriculture) surrounding the mid-point of each transect with 100m, 500m, 1km, 2km, 3km, 4km, and 5km radii buffers. All landcover percentages were arcsine square root transformed for downstream analyses.

Next, we used multi-scale generalized linear mixed models (GLMMs) to determine the appropriate “scale of effect” of landscape characterization on adult tick presence for each tick genus. GLMMs provide a flexible approach for analyzing non-normal data when random effects are present [69]. To determine the scale at which landcover should be considered for each tick genus and landcover type, we used binomial distributions with log link functions in our GLMMs and presence or absence as our response variable. We ran three separate sets of models (one for each focal land-cover) for each adult tick genus with the focal land-cover proportion, ranging from the 100m to 5km scale, included as fixed effects. A random effect for site identity was included in all models. We used the corrected Akaike’s Information Criterion (AICc) for small sample size and model log-likelihoods to select the best scale of effect for each focal landcover [70]. The smallest AICc value indicated the highest quality model, relative to the other models tested. Additionally, we compared the log-likelihood between models, where the model with the highest log-likelihood value is most supported by the data. The land cover types were analyzed separately due to collinearity between land cover proportions. All models were constructed using the lme4 package in R [71].

### Landscape-scale analyses

After determining the appropriate “scale of effect” for savanna, sugarcane, and subsistence agriculture landcover, we quantified additional landscape variables that might influence habitat suitability for ticks. These variables included distance to nearest protected area and distance to nearest homestead which were calculated using Google Earth. Distance to protected area and distance to homestead were square root transformed and z-standardized prior to analysis and landcover proportions were arcsine square root transformed and z-standardized [72].

In our analysis, we first used presence or absence data, from the pooled ten 20m transects, as our response variable, and binomial distributions with log link functions in our GLMMs to run global models for each adult tick genus that included elevation, distance to nearest homestead, and land-cover proportions of savanna, sugarcane agriculture, and subsistence agriculture as fixed effects. Distance to nearest protected area was excluded from this model evaluation because it had a large variance inflation factor (VIF >18) and a strong negative correlation (r = -0.832) with percent savanna cover. We ranked models based on their AICc and considered models that were < 2 AICc units of the best model to be competing models. We used automated model selection to average the best and competing models based on model weight. We considered model parameters with beta estimates and 95% CI that did not include 0 to be relevant predictors. We calculated the conditional R^2^ of the model (R^2^_GLMM(c)_) with the lowest AICc to assess model fit using the MuMIn package in R [73]. The R^2^_GLMM(c)_ is interpreted as the variance explained by the entire model (both fixed and random factors) [74]. Evaluation of tick abundance followed an identical process except we used count data, which was parameterized as the sum of repeated samples across the three days of sampling for all ten 20m transects (200m) in each habitat patch, as our response variable and a negative binomial distribution with log functions in our GLMMs. We calculated the log-normal R^2^_GLMM(c)_ to calculate model variance appropriate for count data [74].

### Patch-scale analyses

We employed a patch-scale adult tick occupancy analysis to identify tick responses to neighborhood effects in their local environment. Subplots within a site were classified into one of four patch categories: 1) in savanna near sugarcane, 2) in savanna near subsistence agriculture, 3) in subsistence agriculture near savanna and 4) in sugarcane near savanna.

We utilized the single-species, single-season occupancy modeling approach [75] to estimate occupancy rates (*psi*) and detection probabilities (*p*) across the study area for adult *H*. *elliptica* and adult *Rhipicephalus* ticks. The detection pattern obtained by surveying subplots across multiple visits within a relatively short time was used to estimate detection probability and correct for false absences [75]. Occupancy modelling uses a general likelihood approach to describe the detection history of a taxon for each visited subplot. Maximum likelihood estimates of the probability of occupancy and probability of detection are derived from the model [75].

For each taxon, the probability of detection was modeled as a function of the survey start time, ambient temperature, relative humidity, and dew point while holding the occupancy component of the model constant. All subplots were included in this initial step of the analysis. Only a single survey-specific covariate was included in each model because of high collinearity assessed using Pearson’s correlation coefficient. Survey-specific covariates were z-standardized prior to occupancy modeling. The best model, according to AICc, of survey-specific covariates for detection probability was selected for each taxon and these covariates were included in subsequent models of site occupancy.

To assess variation in occupancy of each adult tick genus across patch categories, site-specific occupancy covariates of patch landcover classification for each transect were included in occupancy models. We used model selection via the AICc to select the best model that explained variation in occupancy rates for each taxon. The adequacy of model fit for each model was assessed using the MacKenzie-Bailey goodness-of-fit test based on a Pearson’s chi-square statistic [76]. P-values of model fit were generated using 1000 parametric bootstrap simulations [77]. The p-value indicated the proportion of generated data under the fitted model which resulted in a Pearson’s chi-square test statistic greater than or equal to the observed test statistic calculated from empirical data. Small p-values suggest inconsistent fit of the observed data to the fitted model [78]. Finally, parameter estimates across the entire range of covariate values for each model were obtained in the “unmarked” package in R [79]. All analyses were conducted in R statistical software version 3.5.1 [79].

### Edge effect analyses

We used GLMMs to assess edge effects using counts of adult ticks on each of the ten 20-m transects that were perpendicular to the patch edge and extended from the edge to the interior at 10 m intervals (Fig 1). Count data were sufficient only for transects within savanna patches, therefore count data from sugarcane and subsistence agriculture were excluded from these analyses. Tick counts were summed across repeated transects within each subplot (200m total). We ran four separate sets of models using all ticks, total adult ticks, adult *Rhipicephalus*, and adult *H*. *elliptica*. Fixed effects included edge (1 or 0 whether the tick was collected on the edge) and distance from edge; and a random effect for site identity. Candidate-model sets included all combinations of the main effects along with the null model; all models included the random effect. We used negative-binomial distributions with log functions to model our count variables. All model combinations were ranked using the AICc. We predicted abundance estimates to interpret the edge effect of each focal landcover. All models were constructed using the lme4 package in R [71]. We calculated the R^2^_GLMM(c)_ to assess model fit using the MuMIn package in R [73].

## Results

In total, we captured 257 adult, 20 nymphs, and 710 larval ticks over 24 days while sampling a total of 28,800 meters. Of the adult ticks, 65.4% were *H*. *elliptica* (hereafter: *Haemaphysalis*), 12.5% were *R*. *appendiculatus*, 19.8% were *R*. *simus*, and 2.3% were unidentified species in the genus *Rhipicephalus*. Of the nymphal ticks, 20% were *Amblyomma* spp. and 80% were *Rhipicephalus* spp. Larvae occurred in a total of 21 clusters ranging from 1 to over 100 individuals, but were not identified taxonomically. In subsequent analyses, we grouped all adult *Rhipicephalus* ticks to increase sample size. Adult *R*. *appendiculatus* and *R*. *simus* are commonly encountered in similar vegetation types, have similar host preferences (Table 1), and quest for hosts [65].

We detected significantly more ticks in savanna patches than in patches of subsistence agriculture or sugarcane agriculture (chi-squared test: x^2^ = 151.11, df = 2, p-value < 0.001) when using expected values adjusted for sampling effort in each landcover. Specifically, we collected all ticks in fragmented savanna, with the exception of 14 ticks (12 adults and 2 nymphs) in subsistence agriculture and two adults in sugarcane agriculture. We found no evidence of spatial autocorrelation as a function of distance between sites on the abundance of either *Rhipicephalus* or *Haemaphysalis*. Correlations for both genera fell within the null envelope and the p-values of the Monte-Carlo simulation of Moran’s I were insignificant (p = 0.723 for *Rhipicephalus* and p = 0.121 for *Haemaphysalis*).

The ‘scale of effect’ of surrounding landscape characterization on adult tick presence within subplots differed between genera and between focal landcover types. Based on the greatest log-likelihood of the multi-scale generalized linear models, the habitat composition within 100 m was influential for *Rhipicephalus* presence when surveyed in subsistence agriculture and for *Haemaphysalis* when they were surveyed in savanna and sugarcane. The zone of habitat influence was greatest (4-km) for *Rhipicephalus* when they were surveyed in savanna, less (3-km) for *Haemaphysalis* when surveyed in subsistence agriculture, and less still (500-m) for *Rhipicephalus* when surveyed in sugarcane.

### Landscape effects on *Rhipicephalus* and *Haemaphysalis* ticks

**Fig 2:**
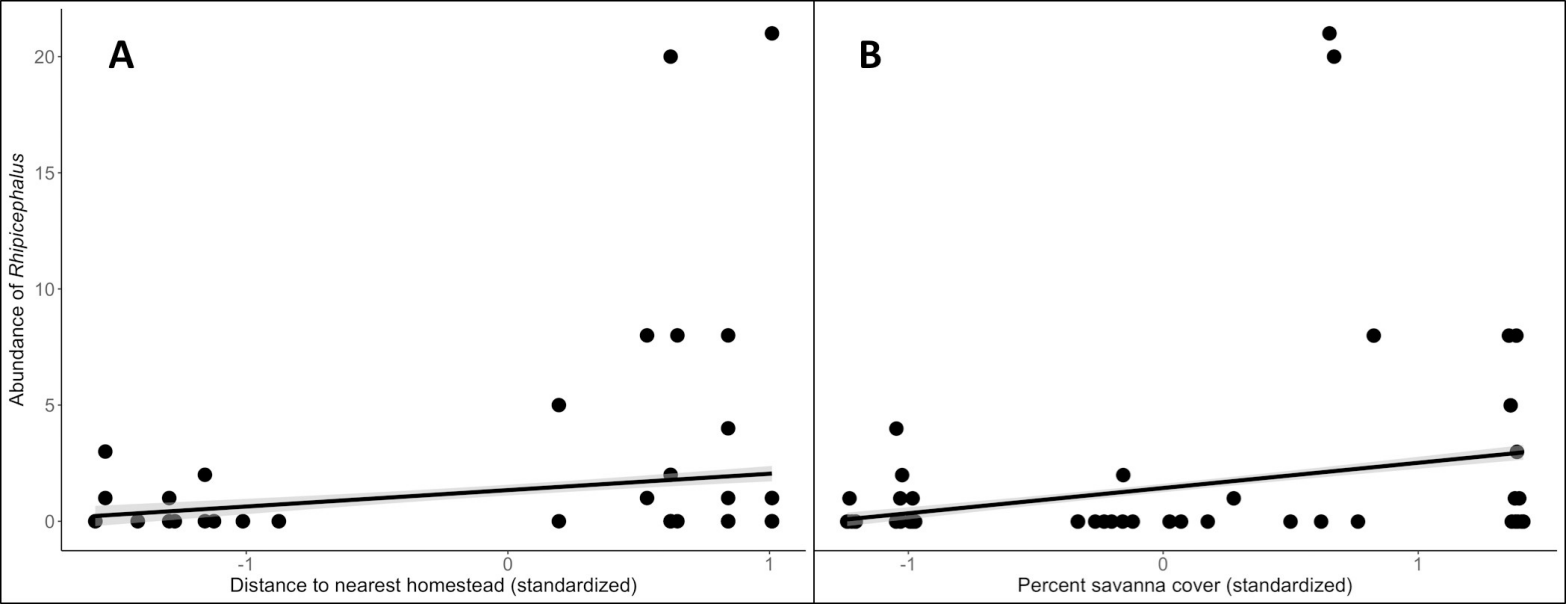
Univariate predictions for the influence of landscape covariates on *Rhipicephalus* ticks. Predictions of *Rhipicephalus* abundance as a function of (A) standardized distance to nearest homestead and (B) standardized percent savanna cover in a 4 km buffer. Actual distance to homestead ranged from 0.04 km to 3.22 km. Actual proportion of savanna land cover ranged from 27.8% to 52.6%.

**Fig 3:**
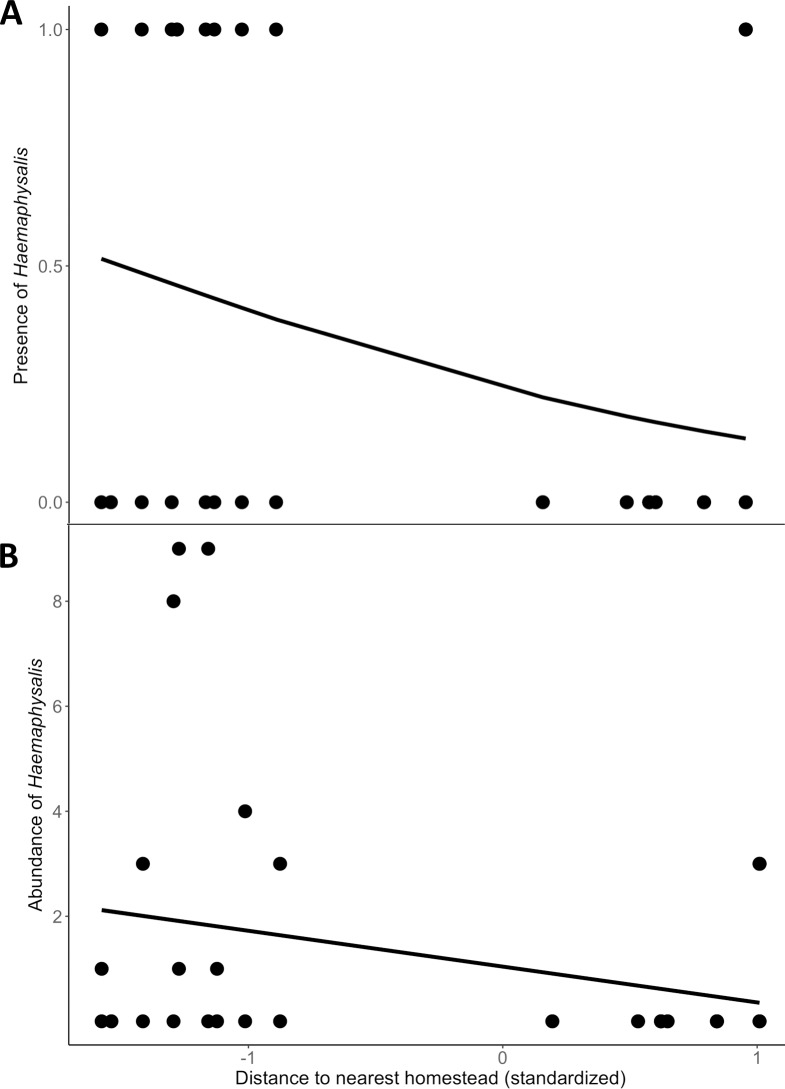
Univariate predictions for the influence of landscape covariates on *Haemaphysalis* ticks. Predictions of *Haemaphysalis elliptica* (A) presence and (B) abundance as a function of standardized distance to nearest homestead. Actual distance to homestead ranged from 0.04 km to 3.22 km.

Landscape features influenced the occupancy of adult *Rhipicephalus* and *Haemaphysalis* in different ways. We found no significant predictors in the model average for occupancy of questing *Rhipicephalus* ticks, however distance to nearest homestead and savanna cover (and therefore distance to nearest protected area) were significant predictors of *Rhipicephalus* abundance (Table 2 and S1 Table). Specifically, locations far from a homestead, close to a protected area, and surrounded by high proportion of savanna cover increased the abundance of *Rhipicephalus* across sites (Fig 2). By contrast, for questing *Haemaphysalis* ticks, proximity to a homestead was a significant predictor of both occupancy and abundance (Table 2) and the model average indicated a strong negative relationship between *Haemaphysalis* and distance to nearest homestead (Fig 3).

**Table 2:** Model average results from top landscape-analysis models of the presence and abundance of questing ticks in the lowveld of Eswatini.

Presence of *Rhipicephalus*						95% Confidence Interval	
	**Estimate**	**Std. Error**	**Adjusted SE**	**z value**	**p value**		
(Intercept)	-0.862	0.370	0.380	2.266	0.023	-1.608	-0.117
Subsistence	-0.896	0.529	0.544	1.647	0.100	-1.962	0.170
Homestead	0.381	0.333	0.343	1.111	0.267	-0.291	1.053
Savanna	0.332	0.324	0.333	0.995	0.320	-0.321	0.985
**Presence of *Haemaphysalis***							
	**Estimate**	**Std. Error**	**Adjusted SE**	**z value**	**p value**		
(Intercept)	-1.131	0.367	0.377	2.996	0.003	-1.870	-0.391
**Homestead**	**-0.779**	**0.348**	**0.358**	**2.176**	**0.030**	**-1.480**	**-0.077**
Savanna	-0.472	0.362	0.372	1.270	0.204	-1.201	0.256
**Abundance of *Rhipicephalus***							
	**Estimate**	**Std. Error**	**Adjusted SE**	**z value**	**p value**		
(Intercept)	-0.295	0.385	0.396	0.747	0.455	-1.071	0.480
Subsistence	-0.970	0.569	0.585	1.658	0.097	-2.117	0.176
**Homestead**	**0.818**	**0.343**	**0.353**	**2.320**	**0.020**	**0.127**	**1.509**
**Savanna**	**0.799**	**0.347**	**0.356**	**2.244**	**0.025**	**0.101**	**1.497**
Elevation	-0.530	0.407	0.418	1.267	0.205	-1.350	0.290
**Abundance of *Haemaphysalis***							
	**Estimate**	**Std. Error**	**Adjusted SE**	**z value**	**p value**		
(Intercept)	-0.281	0.348	0.357	0.787	0.431	-0.981	0.419
**Homestead**	**-0.677**	**0.310**	**0.319**	**2.125**	**0.034**	**-1.302**	**-0.053**
Savanna	-0.694	0.409	0.420	1.652	0.099	-1.517	0.129
Subsistence	-0.587	0.448	0.460	1.277	0.201	-1.489	0.314
Elevation	0.322	0.334	0.344	0.935	0.350	-0.352	0.995

Model covariates: Subsistence = percent cover of subsistence agriculture; Savanna = percent cover of savanna; Homestead = distance to nearest homestead. Bolded covariate predictors are significant. Estimate = parameter estimate; Std. Error = the standard error of parameter estimates, Adjusted SE = the adjusted standard error of parameter estimates.

### Patch-scale tick occupancy

Covariates associated with weather condition at the time of sampling (time of day, temperature, relative humidity, and dew point) did not influence the detection probability of questing *Rhipicephalus* ticks (S2 Table), however, the temperature at time of sampling influenced the detection probability of *Haemaphysalis* (S2 Table). As a result of this effect, all models exploring occupancy covariates for *Rhipicephalus* included no detection covariates, while temperature was included as a detection covariate for all *Haemaphysalis* models, where warmer temperatures increased detection probability. Detection probabilities derived from null (i.e. intercept only) models indicated moderate detection for both taxa (*Rhipicephalus*: p = 0.558, SE = 0.058; *Haemaphysalis*: p = 0.565, SE = 0.067).

**Fig 4:**
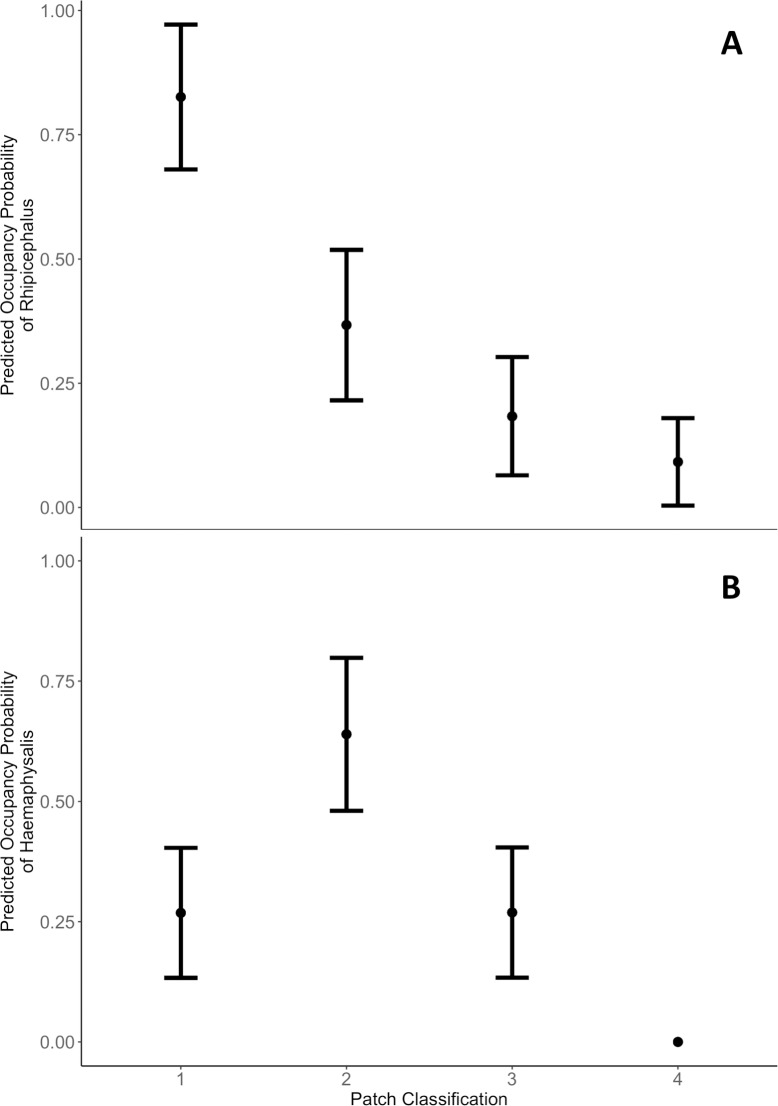
**Predicted occupancy probabilities of (A) *Rhipicephalus* and (B) *Haemaphysalis* adult ticks by patch classification.** Patch classification was defined as ticks collected (1) in savanna near sugarcane, (2) in savanna near subsistence agriculture, (3) in subsistence agriculture near savanna and (4) in sugarcane near savanna. Black points represent mean occupancy values. Error bars represent one standard error.

Occupancy probability was higher within savanna patches for both *Rhipicephalus* and *Haemaphysalis* than in patches of either sugarcane or subsistence agriculture (Fig 4 and S2 Table). However, *Rhipicephalus* and *Haemaphysalis* occupancy was best explained by models that included land use patch neighbor as an occupancy covariate. The highest probability of occurrence for *Rhipicephalus* was in savanna adjacent to sugarcane (psi = 0.826, SE = 0.103), as opposed to *Haemaphysalis* where occupancy was highest in savanna adjacent to subsistence agriculture (psi = 0.636, SE = 0.113) (Fig 4 and S2 Table).

### Edge-effects

We found a significant effect of distance from edge on the abundance of *Rhipicephalus* adults (p = 0.0282), but not for *Haemaphysalis* adults (p = 0.6481), combined adults of both species (p = 0.464), or total abundance of ticks (p = 0.113) (S3 Table). The predicted occurrence of *Rhipicephalus* was greater farther from the patch edge (estimate = 0.2543, SE = 0.1159, *z*-score = 2.194, *p*-value = 0.028, R^2^_GLMM(c)_ = 0.540). No models that included a binary covariate for the presence or absence of ticks collected on transects at the edge of a patch vs. transects more interior in the savanna patch were significant (S3 Table).

## Discussion

In a region with rapidly changing land uses, understanding the landscape ecology of tick communities in the lowveld of southern Africa provides important information about risks to human, domestic animal and wildlife health. Our study provided insight into how two common genera of ticks found in southern Africa utilize the landscape differently. Those differences were apparent at multiple spatial scales, and showed that life history traits influenced distribution patterns in contrasting ways.

Several landscape scale factors influenced adult tick occupancy and abundance and the direction of effects differed among *Rhipicephalus* and *Haemaphysalis* ticks. Following our expectations on host availability and host preference, *Rhipicephalus* ticks occurred at significantly higher abundances when the savanna site was surrounded by a high percentage of additional savanna cover, close to a protected area, and far from a homestead. These are characteristics of habitats that contain or facilitate movement of medium to large wildlife hosts. Additionally, *Haemaphysalis* occurred in significantly higher abundances when the site was closer to a homestead. *Haemaphysalis elliptica* is a canid specialist and therefore its distribution likely matches the occurrence patterns of dogs that live in and near homesteads. The sole similarity in our landscape models between the two genera was the general decrease in abundance as subsistence agriculture landcover increased (Table 2). This trend may be a result of 1) fewer hosts in the vicinity when subsistence agriculture is the dominant land cover, 2) subsistence agriculture does not provide a suitable microclimate when ticks are off host, or 3) subsistence agriculture provides less appropriate vegetation from which to quest.

Both *Rhipicephalus* and *Haemaphysalis* ticks occupied savanna patches more reliably than other habitat types. Overall, approximately 95% of adult ticks detected occurred in savanna fragments. This result may reflect differences in (1) host species availability, (2) suitable microhabitats within each land cover type or (3) land management such as burning that occurs most frequently in sugarcane [80]. Despite the more frequent occurrence of ticks in savanna, neighboring habitat classification produced genus-specific differences in questing ticks. Specifically, when subsistence agriculture, which contains domestic animals such as dogs [10, 11], was next to savanna patches, it appeared to increase the abundance of *Haemaphysalis* in those savanna patches. When savanna fragments were next to sugarcane patches and likely further away from homesteads, the observed increase in adult *Rhipicephalus* ticks may be due to a higher number of wild ungulate hosts that can utilize sugarcane or received less hunting pressure [81, 82].

At the patch scale, *Rhipicephalus* adults were more likely to occur in greater numbers towards the interior of a savanna patch than on the patch edge. *Haemaphysalis* adults did not show any trends in abundance around the interface of land-uses. These results did not match our predictions of increased tick abundance at edges due to edge-related increases in species abundance or diversity that occur when species can utilize resources from multiple habitats or when adjoining land-uses provide novel resources [83]. Our results add to mixed findings on edge-effects for ticks. Several studies in Europe and North America have shown tick vectors are more abundant near habitat edges [36, 84], where they may encounter a wider range of potential hosts [48] or a higher density of suitable hosts [36]. At these edges, generalist species that utilize multiple land uses may increase in abundance, while species that require resources from only one land use may be more abundant at the core, or further away from the edge [85, 86]. However, we found evidence that *Rhipicephalus* adults avoided savanna edges which has previously been found for other species of ticks in forested habitats [87, 88]. Edges can be associated with decreased quality for some species, such as habitat specialists, who are negatively impacted by proximity to non-habitat [83, 89]. In our study, vertebrate hosts of *Rhipicephalus* ticks could be negatively impacted by edges while the hosts of *Haemaphysalis* ticks were not. Alternatively, differences in microclimate could exist between edges and the interior of a patch, resulting in edges being drier and making ticks more prone to desiccation. Ultimately, we did not find that edges increased the probability of a tick encounter compared to the interior of a savanna patch, although encounter risk at savanna edges was still greater than in subsistence or sugarcane agricultural habitat.

Landscape processes operate on a variety of spatial scales, and there is ongoing uncertainty about the appropriate scale for studying a particular ecological process [68]. Our results indicate that the scale of geographic influence differs among tick genera, and that focal landcover types differ in their scale of influence within each genus. These complex interactions between tick taxa and habitat heterogeneity suggest that future studies on the landscape ecology of ticks should take into account different spatial scales, as important processes may differ across scales.

The use of occupancy modeling for estimating species provides a more accurate accounting of species that are detected imperfectly, such as species that are sensitive to weather conditions and may go undetected during suboptimal weather [75]. We considered the potential problem of weather-related variation in genera detection probability by resampling transects on multiple days and incorporating those repeated measures into single-season single-species occupancy models. Surprisingly, estimates of occupancy were not improved by incorporating any survey-specific covariates into the model for adult *Rhipicephalus*, but incorporating temperature improved the model for *Haemaphysalis* ticks. This result highlights genus-specific differences in detectability as a response to weather-related variation. The lack of model improvement using occupancy modeling for *Rhipicephalus* may have been a product of different host seeking behaviors or of our restrictive sampling protocol that limited surveys to the morning hours shortly after the vegetation had dried from the morning dew.

All tick species included in this study are three-host species that generally complete one life cycle per year in the field [10], however notable differences exist between species in their questing behavior and seasonal occurrence which may influence their ability to be effectively sampled using flagging methods during a restricted time of year. All stages of development of *R*. *appendiculatus* quest for hosts from the vegetation [90, 91]. Due to the tendency of *R*. *simus* larvae and nymphs to feed on rodents, *R*. *simus* larvae and nymphs quest from the soil surface, the lowest vegetation layer, or rodent nests or burrows. Unfed adult *R*. *simus* quest for their larger hosts from the soil surface or surrounding vegetation [92, 93]. Adult *Haemaphysalis elliptica* quest for their carnivore hosts from the vegetation [92, 94]. The immature stages of *H*. *elliptica* are rarely collected from the vegetation [91, 95] because they quest for rodent hosts from the soil surface or from rodent nests and burrows [96]. Despite the detection of nymphal *Amblyomma* at our site, and the occurrence of adult *A*. *hebraeum* crawling on humans at our field sites during surveys, it was unsurprising that no adult *A*. *hebraeum* were collected during our flag sampling. The larvae of *A*. *hebreaum* quest for hosts from the vegetation [97, 98] and can be collected in large quantities by dragging or flagging [98]. The nymphs and adults of *A*. *hebreaum* quest from the soil surface and leaf litter and therefore are rarely collected by dragging in vegetation [99].

Variable seasonal occurrence may also influence the detection of ticks and their abundance in a specific location at a given time of year, and this poses limitation in the interpretation of our study outside of the austral winter season. Despite this, our study does provide important information about the habitat preferences of *Rhipicephalus* and *Haemaphysalis* ticks and the influence of landscape on tick occupancy and abundance during the dry winter. While seasonal trends of life stages do exist for the species in this study, there are differences in the duration and peak period of activity among years and sites that reflective of differences in local climatic conditions and year-to-year variations in temperature and rainfall, as well as changes in host density and habitat use [91]. While biases exist in our sampling methodology, we believe they were applied equally to all habitat types, and thus our results adequately reflect differences in the number of questing ticks among different habitat types and within savanna patches reflect differences in tick abundance due to landscape and local processes (via host and vegetation variation).

This study complements a growing body of literature that suggests land cover and land use are important variables when describing the fine-scale distribution of ticks and tick-borne diseases [45, 100–102]. By using a multi-scale modeling approach, we found that tick genera responded to a fragmented, agricultural landscape in ways that were consistent with their host preferences. *Rhipicephalus* ticks in this study system include those that are capable vectors of human pathogens in the spotted fever group (*Rickettsia aeschlimanni* and *Rickettsia conorii conorii*) and livestock pathogens including *Theileria* spp. and *Anaplasma* sp. (Table 1). This genus of ticks occurred in higher abundance near conservation areas where wild ungulates remain. *H*. *elliptica* ticks, which can transmit the agent of Mediterranean spotted fever to humans (*Rickettsia conorii conorii*) and canine babesiosis (*Babesia canis*) to dogs, occurred in higher abundance next to homesteads where large numbers of domestic animals are found.

In summary, our results show the composition and configuration of land cover types at the landscape, neighborhood, and local scale influence the occurrence of ticks. The increase in high intensity agriculture and associated savanna habitat fragmentation may lead to a concentration of hosts into protected areas, which in turn may promote increased transportation of ticks and transmission of pathogens [103]. These landscape level processes may therefore drive disease emergence as natural ecosystems become converted to agriculture and remaining savanna is confined to protected areas. Emergence or re-emergence of many pathogens is due to the disruption of stable ecosystems as disease processes are altered and new host species are incorporated into the epidemiological process [104]. The creation of novel ecosystems and epidemiological landscapes has unknown consequences for the future of human and animal health.

It is timely to understand the ecosystem-wide implications of agricultural intensification and the potential risks to animals and humans in these settings as land use change continues and may contribute to the emergence of additional zoonotic and livestock pathogens over time. The way in which habitats are managed on a landscape scale, and the changes in distribution and abundance of ticks are important considerations as the southern African landscape continues to undergo major shifts in agricultural land use. The increase in high intensity agriculture and associated savanna habitat fragmentation may lead to a concentration of hosts into savanna areas which supports an increased concentration of ticks and transmission of pathogens.

## Supporting information

S1 Table Landscape effects on tick genera. Top model selection results (delta AICc < 2) for models evaluating the effects of distance to homestead, percent savanna cover, percent sugarcane cover, and percent subsistence agriculture cover on (A) *Rhipicephalus* presence, (B) *Haemaphysalis* presence, (C) *Rhipicephalus* abundance, (D) *Haemaphysalis* abundance. R^2^_GLMM(c)_ indicates the conditional model fit.
(DOCX)

S2 Table Occupancy modeling. Model selection table for A) *Rhipicephalus* detection, B) *Rhipicephalus* occupancy, C) *Haemaphysalis* detection, D) *Haemaphysalis* occupancy. ΔAICc is the relative difference in AICc values from the model with the smallest AIC value; *w* is the AIC model weight; *K* is the number of parameters, ψ-hat is the estimated overall occupancy probability; SE(ψ*-*hat) is the associated standard error for the estimate; *X* ^2^ is the test statistic for model fit; *p* value is the probability of observing a test statistic ≥ *X* ^2^ based upon 1000 parametric bootstraps; and *c*-hat is the estimated overdispersion parameter.
(DOCX)

S3 Table Edge effects. Effects of distance to savanna patch edge (Distance) and being located at the patch edge (Edge) from GLMMs on the number of total ticks, total adult tick, *Rhipicephalus* adult abundance, and *Haemaphysalis* adult abundance. Fixed effects for all models included Site. Bolded rows indicate statistical significance. K is the number of parameters, ΔAICc is the relative difference in AICc values from the model with the smallest AIC value, ML is the model likelihood, *w* is the AIC model weight; LL is the log-likelihood; and R^2^_GLMM(c)_ indicates the conditional model fit. P-value of the intercept, distance, and edge covariates are also reported for each model.
(DOCX)
