# Supplementary material for: Multi-scale patterns of tick occupancy and abundance across an agricultural landscape in southern Africa
Source: PLoS One. 2019 Sep 20;14(9):e0222879. doi: 10.1371/journal.pone.0222879 (PMC6754170; doi:10.1371/journal.pone.0222879)
Supplement: S1 Table — Top model selection results (delta AICc < 2) for models evaluating the effects of distance to homestead, percent savanna cover, percent sugarcane cover, and percent subsistence agriculture cover on (A) Rhipicephalus presence, (B) Haemaphysalis presence, (C) Rhipicephalus abundance, (D) Haemaphysalis abundance. R2GLMM(c) indicates the conditional model fit. (DOCX) [file pone.0222879.s001.docx]

| **(Intercept)** | **Subsistence** | **Elevation** | **Homestead** | **Savanna** | **Sugarcane** | **df** | **logLik** | **AICc** | **delta AICc** | **weight** | **R^2^_GLMM(c)_** |
| --- | --- | --- | --- | --- | --- | --- | --- | --- | --- | --- | --- |
| **A) Presence of *Rhipicephalus*** | | |  |  |  |  |  |  |  |  |  |
| -0.857 | -0.919 | NA | NA | NA | NA | 3 | -28.081 | 62.708 | 0.000 | 0.401 | 0.206 |
| -0.870 | -0.936 | NA | 0.381 | NA | NA | 4 | -27.401 | 63.732 | 1.024 | 0.240 | 0.225 |
| -0.863 | -0.806 | NA | NA | 0.332 | NA | 4 | -27.546 | 64.022 | 1.314 | 0.208 | 0.218 |
| -0.890 | -0.815 | NA | 0.461 | 0.421 | NA | 5 | -26.610 | 64.649 | 1.941 | 0.152 | 0.255 |
| **B) Presence of *Haemaphysalis*** | | |  |  |  |  |  |  |  |  |  |
| -1.109 | NA | NA | -0.742 | NA | NA | 3 | -25.486 | 57.517 | 0.000 | 0.437 | 0.145 |
| -1.160 | NA | NA | -0.829 | -0.472 | NA | 4 | -24.580 | 58.090 | 0.573 | 0.328 | 0.198 |
| -1.144 | NA | 0.358 | -0.782 | NA | NA | 4 | -24.917 | 58.765 | 1.248 | 0.234 | 0.181 |
| **C) Abundance of *Rhipicephalus*** | | |  |  |  |  |  |  |  |  |  |
| -0.378 | -1.023 | NA | 0.833 | 0.785 | NA | 6 | -63.217 | 140.483 | 0.000 | 0.372 | 0.604 |
| -0.213 | NA | -0.627 | 0.780 | 0.783 | NA | 6 | -63.991 | 142.031 | 1.548 | 0.171 | 0.496 |
| -0.081 | NA | NA | 0.877 | 0.967 | NA | 5 | -65.369 | 142.167 | 1.683 | 0.160 | 0.453 |
| -0.408 | -0.846 | -0.425 | 0.765 | 0.678 | NA | 7 | -62.695 | 142.190 | 1.707 | 0.158 | 0.607 |
| -0.176 | NA | -1.510 | NA | NA | 1.042 | 5 | -65.517 | 142.463 | 1.980 | 0.138 | 0.552 |
| **D) Abundance of *Haemaphysalis*** | | |  |  |  |  |  |  |  |  |  |
| -0.171 | NA | NA | -0.645 | NA | NA | 4 | -56.844 | 122.618 | 0.000 | 0.236 | 0.176 |
| -0.295 | NA | NA | -0.660 | -0.585 | NA | 5 | -55.616 | 122.660 | 0.042 | 0.231 | 0.270 |
| -0.445 | -0.691 | NA | -0.758 | -0.807 | NA | 6 | -54.350 | 122.749 | 0.131 | 0.221 | 0.436 |
| -0.224 | -0.385 | NA | -0.713 | NA | NA | 5 | -56.330 | 124.089 | 1.471 | 0.113 | 0.287 |
| -0.219 | NA | 0.322 | -0.584 | NA | NA | 5 | -56.370 | 124.168 | 1.550 | 0.109 | 0.182 |
| -0.174 | NA | NA | NA | NA | NA | 3 | -58.984 | 124.513 | 1.895 | 0.091 | 0 |
