# Supplementary material for: Multi-scale patterns of tick occupancy and abundance across an agricultural landscape in southern Africa
Source: PLoS One. 2019 Sep 20;14(9):e0222879. doi: 10.1371/journal.pone.0222879 (PMC6754170; doi:10.1371/journal.pone.0222879)
Supplement: S2 Table — Model selection table for A) Rhipicephalus detection, B) Rhipicephalus occupancy, C) Haemaphysalis detection, D) Haemaphysalis occupancy. ΔAICc is the relative difference in AICc values from the model with the smallest AIC value; w is the AIC model weight; K is the number of parameters, ψ-hat is the estimated overall occupancy probability; SE(ψ-hat) is the associated standard error for the estimate; X 2 is the test statistic for model fit; p value is the probability of observing a test statistic ≥ X 2 based upon 1000 parametric bootstraps; and c-hat is the estimated overdispersion parameter. (DOCX) [file pone.0222879.s002.docx]

| **Models** | **ΔAICc** | ***w*** | **K** | ψ***-*hat** | **SE(**ψ***-*hat)** | ***X*^2^** | ***p* value** | ***c*-hat** |
| --- | --- | --- | --- | --- | --- | --- | --- | --- |
| **A. *Rhipicephalu*s Detection** | | | | | | | | |
| ψ(·)*p*(·) | 0 | 0.4168 | 2 | 0.367 | 0.0779 | 3.9206 | 0.582 | 0.7853 |
| ψ(·)*p*(dew) | 1.9565 | 0.1567 | 3 | 0.365 | 0.0772 | 4.3384 | 0.524 | 0.8632 |
| ψ(·)*p*(temp) | 2.0918 | 0.1464 | 3 | 0.366 | 0.0775 | 3.9974 | 0.565 | 0.7994 |
| ψ(·)*p*(time) | 2.1154 | 0.1447 | 3 | 0.366 | 0.0776 | 3.813 | 0.601 | 0.7542 |
| ψ(·)*p*(RH) | 2.2486 | 0.1354 | 3 | 0.367 | 0.0779 | 4.0018 | 0.565 | 0.7985 |
| **B. *Rhipicephalus* Occupancy** | | | | | | | | |
| ψ(Patch)*p*(·) | 0 | 0.9800 | 5 | See Fig4 | See Fig4 | 3.9206 | 0.604 | 0.7727 |
| ψ(·)*p*(·) | 7.47 | 0.0200 | 2 | 0.367 | 0.0779 | 3.9206 | 0.582 | 0.7853 |
| **C. *Haemaphysalis* Detection** | | | | | | | | |
| ψ(·)*p*(temp) | 0 | 0.8643 | 3 | 0.292 | 0.0707 | 4.4095 | 0.488 | 0.9062 |
| ψ(·)*p*(RH) | 4.6571 | 0.9485 | 3 | 0.290 | 0.0700 | 4.1464 | 0.525 | 0.8349 |
| ψ(·)*p*(·) | 6.7843 | 0.0291 | 2 | 0.295 | 0.0720 | 5.1076 | 0.432 | 0.9541 |
| ψ(·)*p*(dew) | 8.3957 | 0.0130 | 3 | 0.299 | 0.0734 | 4.6006 | 0.493 | 0.9069 |
| ψ(·)*p*(time) | 9.0287 | 0.0095 | 3 | 0.296 | 0.0723 | 5.1755 | 0.364 | 1.0453 |
| **D. *Haemaphysalis* Occupancy** | | | | | | | | |
| ψ(Patch)*p*(temp) | 0 | 0.9398 | 6 | See Fig4 | See Fig4 | 4.915 | 0.407 | 1.028 |
| ψ(·)*p*(temp) | 5.4960 | 0.0603 | 3 | 0.292 | 0.0707 | 4.4095 | 0.488 | 0.9062 |
