# Supplementary material for: Multi-scale patterns of tick occupancy and abundance across an agricultural landscape in southern Africa
Source: PLoS One. 2019 Sep 20;14(9):e0222879. doi: 10.1371/journal.pone.0222879 (PMC6754170; doi:10.1371/journal.pone.0222879)
Supplement: S3 Table — Effects of distance to savanna patch edge (Distance) and being located at the patch edge (Edge) from GLMMs on the number of total ticks, total adult tick, Rhipicephalus adult abundance, and Haemaphysalis adult abundance. Fixed effects for all models included Site. Bolded rows indicate statistical significance. K is the number of parameters, ΔAICc is the relative difference in AICc values from the model with the smallest AIC value, ML is the model likelihood, w is the AIC model weight; LL is the log-likelihood; and R2GLMM(c) indicates the conditional model fit. P-value of the intercept, distance, and edge covariates are also reported for each model. (DOCX) [file pone.0222879.s003.docx]

| **Model** | **K** | **ΔAICc** | **ML** | ***w*** | **LL** | **R^2^_GLMM(c)_** | **Fixed effects: p-values** | | |
| --- | --- | --- | --- | --- | --- | --- | --- | --- | --- |
|  |  |  |  |  |  |  | **Intercept** | **Distance** | **Edge** |
| **Total Ticks** |  |  |  |  |  |  |  |  |  |
| Distance | 4 | 0 | 1 | 0.355 | -343.596 | 0.284 | 0.824 | 0.113 | NA |
| Null | 3 | 0.372 | 0.830 | 0.295 | -344.816 | 0.287 | 0.855 | NA | NA |
| Edge | 4 | 1.054 | 0.591 | 0.210 | -344.123 | 0.290 | 0.298 | NA | 0.239 |
| Distance:Edge | 5 | 1.845 | 0.398 | 0.141 | -343.475 | 0.286 | 0.883 | 0.265 | 0.251 |
| **Adult Ticks** |  |  |  |  |  |  |  |  |  |
| Null | 3 | 0 | 1 | 0.497 | -305.600 | 0.382 | 0.273 | NA | NA |
| Distance | 4 | 1.554 | 0.460 | 0.229 | -305.343 | 0.377 | 0.263 | 0.464 | NA |
| Edge | 4 | 1.894 | 0.388 | 0.193 | -305.513 | 0.380 | 0.293 | NA | 0.674 |
| Distance:Edge | 5 | 3.632 | 0.163 | 0.081 | -305.339 | 0.377 | 0.272 | 0.671 | 0.55 |
| ***Rhipicephalus* Ticks** | |  |  |  |  |  |  |  |  |
| **Distance** | **4** | **0** | **1** | **0.485** | **-163.807** | **0.540** | **< 0.01** | **0.028** | **NA** |
| Distance:Edge | 5 | 1.595 | 0.450 | 0.219 | -163.562 | 0.545 | < 0.01 | 0.157 | 0.109 |
| Edge | 4 | 2.071 | 0.355 | 0.172 | -164.843 | 0.544 | < 0.01 | NA | 0.125 |
| Null | 3 | 2.727 | 0.256 | 0.124 | -166.205 | 0.533 | < 0.01 | NA | NA |
| ***Haemaphysalis* Ticks** | |  |  |  |  |  |  |  |  |
| Null | 3 | 0 | 1 | 0.525 | -217.161 | 0.552 | 0.016 | NA | NA |
| Distance | 4 | 1.867 | 0.393 | 0.206 | -217.060 | 0.557 | 0.017 | 0.648 | NA |
| Edge | 4 | 1.975 | 0.372 | 0.196 | -217.114 | 0.554 | 0.105 | NA | 0.758 |
| Distance:Edge | 5 | 3.943 | 0.139 | 0.073 | -217.055 | 0.557 | 0.017 | 0.765 | 0.728 |
